# Measurement and Characterization of the Electrical Properties of Actin Filaments

**DOI:** 10.3390/ijms25105485

**Published:** 2024-05-17

**Authors:** Serena Paladini, Barbara Truglia, Karthik Shankar, Jack Adam Tuszynski

**Affiliations:** 1 Department of Mechanical and Aerospace Engineering (DIMEAS), Politecnico di Torino, Corso Duca degli Abruzzi 24, 10129 Torino, Italy; serenapaladini97@gmail.com (S.P.); truglia@ualberta.ca (B.T.); 2 Department of Electrical and Computer Engineering, University of Alberta, Edmonton, AB T6G 2R3, Canada; kshankar@ualberta.ca; 3 Department of Physics, University of Alberta, Edmonton, AB T6G 2E1, Canada; 4 Department of Data Science and Engineering, The Silesian University of Technology, 44-100 Gliwice, Poland

**Keywords:** actin, cytoskeleton, actin polymerization, nano-biowires, electrical properties of biomacromolecules

## Abstract

Actin filaments, as key components of the cytoskeleton, have aroused great interest due to their numerous functional roles in eukaryotic cells, including intracellular electrical signaling. The aim of this research is to characterize the alternating current (AC) conduction characteristics of both globular and polymerized actin and quantitatively compare their values to those theoretically predicted earlier. Actin filaments have been demonstrated to act as conducting bionanowires, forming a signaling network capable of transmitting ionic waves in cells. We performed conductivity measurements for different concentrations of actin, considering both unpolymerized and polymerized actin to identify potential differences in their electrical properties. These measurements revealed two relevant characteristics: first, the polymerized actin, arranged in filaments, has a lower impedance than its globular counterpart; second, an increase in the actin concentration leads to higher conductivities. Furthermore, from the data collected, we developed a quantitative model to represent the electrical properties of actin in a buffer solution. We hypothesize that actin filaments can be modeled as electrical resistor–inductor–capacitor (RLC) circuits, where the resistive contribution is due to the viscous ion flows along the filaments; the inductive contribution is due to the solenoidal flows along and around the helix-shaped filament and the capacitive contribution is due to the counterion layer formed around each negatively charged filament.

## 1. Introduction

Since Straub’s discovery of actin in muscle tissue in 1942 [1], actin has been demonstrated to be the most abundant cytoskeletal protein in many eukaryotic cells [2]. Actin performs a wide range of crucial cellular functions, including its pivotal role in cell motility and tracking the transport of motor protein complexes. It exists in two main forms: monomeric G-actin and polymerized F-actin, co-present in a dynamic equilibrium inside the cytoskeleton. One adenosine-like nucleotide is tightly bound to actin, and the G- to F-actin polymerization activates the ATPase, having rates of polymerization and depolymerization dependent on the concentrations of free actin, ATP and ADP [3].

Actin, in its monomeric form, is a globular protein with a molecular weight of 42 kDa and roughly 5.5 nm in diameter [4]. It consists of a single polypeptide chain of 375 amino acids, globally acidic. The filaments of actin, denoted as F-actin, are linear chains of actin monomers organized in two helical strands that are twisted around each other to form a 7 to 9 nm diameter helix that repeats every 72 nm [2]. When in the presence of a nucleotide and a divalent cation at physiological salt concentrations, actin monomers can spontaneously polymerize. The filament’s polarity manifests itself by the presence of two different solvent-exposed surfaces of the actin monomers as they are progressively added to both ends of the filament. The leading end is called the barbed (or plus) end, while the trailing end is referred to as the pointed (or minus) end [5]. New actin monomers preferentially attach to the plus end of an existing actin filament while shortening by preferential detachment of monomers from the minus end, although both ends can grow and shorten, albeit at different rates that depend on both actin and ATP concentrations. This behavior is also known as the “actin assembly dynamic” [6].

Under physiological conditions, actin filaments represent highly charged polyelectrolytes [7] that can be modeled as infinitely long cylinders with a distribution of charges along their surface [8,9,10]. Such an approximation is, in general, quite versatile and can be applied even in the case of coiled macro-ions [8,11]. Following the condensation theory proposed by Manning [12,13], under the hypothesis of infinitely diluted solutions, there exists a critical charge density at which polyelectrolytes may attract condensed ions to their surroundings. These ions are called counterions, and condense along the length of the polymer, reducing the net charge density in the process. The idea of counterion condensation was first introduced by Oosaka and, together with the aforementioned Manning theory, it defines the Debye length as the distance representing the interaction range between counterions and co-ions present in a salt solution [8,11,12,13]. The Debye length is given by the formula [14,15]

$$\lambda_{D}=\sqrt{(\varepsilon_{0}\varepsilon_{r}k_{B}T)/(2N_{A}c_{s}e^{2}})$$

where $\varepsilon_{0}$ is the electric permittivity of vacuum, $\varepsilon_{r}$ is the dielectric constant of the medium, *k_B_* is the Boltzmann constant, *T* the absolute temperature, *N_A_* Avogardo’s number, and *c_s_* is the solvent ion concentration. Moreover, it can be considered as a measure of the screening by counterions of very large, charged macromolecules in a solution, such as DNA [14,16].

Manning stated that the total sum of charges on the surface is proportional to the valence of ions and to the Bjerrum length that was extrapolated by Bjerrum himself from the Debye–Hückel limiting law, considering small ions, a small dielectric medium constant and a high valence [12,17,18]. The Bjerrum length is equal to

$$l_{B}=\frac{e^{2}}{\left(\varepsilon k_{B}T\right)},$$

and it represents the distance at which the electrostatic interaction between two elementary charges *e* in a medium having dielectric constant ε equals the thermal energy [19].

Also, the interactions between the cylinder, the condensed counterions and small ions present in the solution as uncondensed can be analyzed using the Debye–Hückel approximation [14,15,20,21,22]. The latter is a linearization of the Poisson–Boltzmann model but only considers small potentials in electrolytes that are relatively diluted. This approximation defines the formation of an electrical double layer (EDL) of ions condensing on the polyelectrolyte solution, generating a sort of ionic atmosphere dependent on such length, whose thickness is directly proportional to the dielectric constant of the solution itself [23]. Considering the case of physiological conditions, the double layer is formed by a nonuniform positioning of counterions and co-ions, whose interaction can be modeled by a combination of ionic conductivity (resistor) and a capacitance layer intrinsically related to the EDL [14,24,25].

Specifically for actin filaments, there are highly negative charges present on their surfaces such that the counterion layer is formed in the saline solution with co-ions repelled from the filament’s vicinity. Functionally, actin polymers have been modeled as nonlinear inhomogeneous electrical transmission “cables” and are shown to produce nonlinear ionic solitary waves representing electrical signals that propagate with virtually no loss of energy along their path [26]. However, the underlying biophysical principles and molecular mechanisms that support the ionic conductance and transport along actin filaments are still inadequately understood. 

Recently, diverse multi-scale methods in modeling infinitely long cylindrical filaments in electrolyte solutions have been developed to provide insights into the interactions along the EDL. One of these in particular has been proven to accurately represent the complex effects of the diffuse part of the EDL on ionic conductivity and capacitance [25]. These contributions resulted in being highly influenced by the width of the EDL, the electrical surface potential of the filament, the strength of the ions and the composition of the electrolyte fluid [27]. 

Furthermore, another multi-scale approach has been proposed by Hunley et al. in which instead of an EDL representation which is usually employed, a nonlinear Debye–Hückel approximation has been applied to a triple-layer structure [28]. Such an approximation is the generalization of the classical Debye–Hückel model, and it allows one to express the mean electrostatic potential in an accurate way as a function of the effective surface charge density while being consistent with Manning’s theory [29,30]. This new model splits the EDL into two condensation regions: one contains only multivalent counterions, and the second one represents a diffuse layer where both counterions and co-ions are present [28]. However, while computational modeling of the conductive properties of actin filaments has resulted in several publications, the corresponding literature on the experimental determination of actin conductivity is relatively sparse. This has given us the motivation to investigate in detail the conditions allowing actin filaments to act as electrical transmission lines for ion fluxes propagating along their length. 

The aim of this research is to shed light on the electrical properties of actin filaments, in particular considering the AC characteristics. Hence, this study represents a novelty for the whole research community interested in actin and bioelectricity for three main reasons: firstly, only computational models have been developed, but there is a lack of experimental data apart from predictions; secondly, such computational evidence focuses mainly on giving actin a pulse as input rather than AC conductive currents; finally, the comparison between G- and F-actin is not existent in the literature. Indeed, taking into consideration the above background, following a reconstitution of the actin protein and a successful polymerization, impedance values of the sample solutions were measured. The results of such measurements were then compared to both the background and G-actin-containing solutions. Based on the analysis of the results, a model has been created to characterize the actin filament properties, in which each filament is modeled by an electric element having capacitive, inductive and resistive characteristics due to its molecular structure and the viscosity of the solution. Finally, the leading hypothesis adopted in our study is the presence of percolated resistors and capacitors within the circuit due to the complexity of the organization of such filaments in the samples subjected to electrical potential gradients.

## 2. Results

### 2.1. Turbidity Measurements

**Figure 1:**
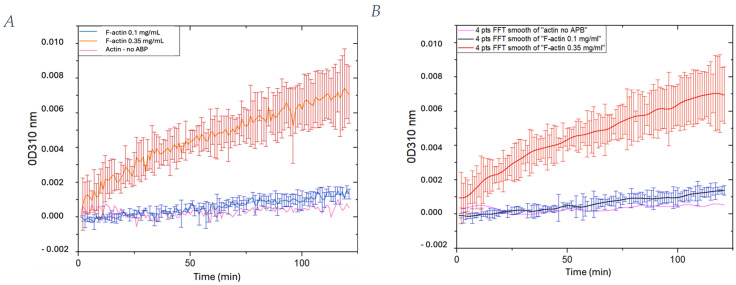
Standard polymerization reactions of samples of actin at different concentrations: 0.1 mg/mL and 0.35 mg/mL. A control solution without the addition of actin polymerization buffer has been analyzed as well. (**A**) Data obtained; (**B**) data smoothed with FFT windows of 4 points.

Through the use of an actin polymerization assay, it is possible to assess the biological functionality of AKL99 (actin protein purified from rabbit skeletal muscle, 99% purity). The ability of globular actin to polymerize into filaments can be observed from the increase in optical density of actin solution at OD 310 nm (Figure 1). Polymerization assays were carried out at 25 °C with the SpectraMax^®^ iD5 microplate reader (Molecular Devices LLC, San Jose, CA, USA), as indicated in the Materials and Methods section. First, for F-actin, two different concentrations were analyzed (0.1 mg/mL and 0.35 mg/mL): the polymerization in the latter case is more evident, showing a linear trend. It was also smoothed with a 4-points-window Fast Fourier Transform (Figure 1B). In both cases, there was no evidence of the classical assembly in three steps (nucleation phase, elongation phase and steady-state phase). As a matter of fact, such an assembly is commonly due to the involvement of proteins such as actin-related protein 2/3 complex (Arp2/3) or verprolin–cofilin–acidic domain (VCA) but in the present case, they were not included in the experimental solutions.

### 2.2. Electrical Conductance Measurements

**Figure 2:**
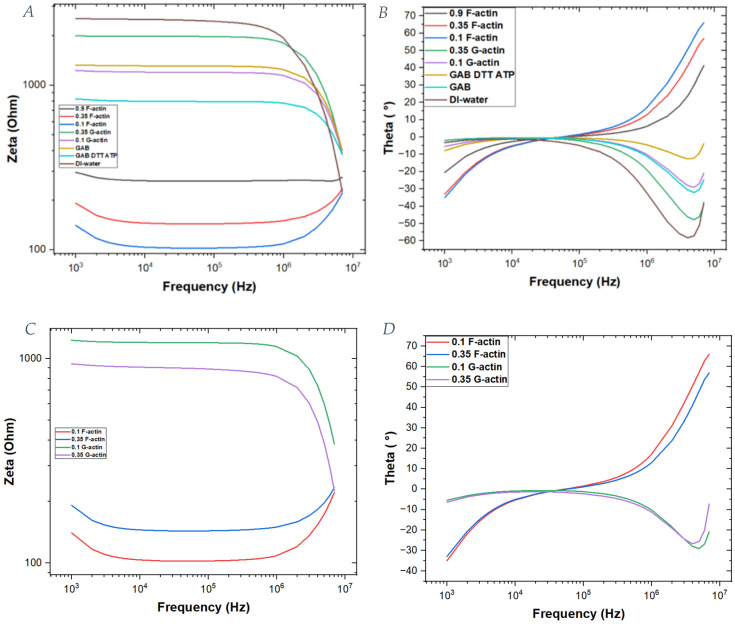
Impedance (zeta) and phase (theta) of an actin sample and buffer solutions in the frequency range between 103 and 107 Hz. (**A**) Impedance results of the samples. (**B**) Phase trend of the samples. (**C**) Focus on the impedance of actin filaments and globular actin at two different concentrations. (**D**) Focus on the phase of actin filaments and globular actin.

From Figure 2, we infer the absolute values of impedance and phase for the actin filaments, globular actin solutions and their background. In the case of F-actin, a third concentration equal to 0.9 mg/mL has also been analyzed. 

**Figure 3:**
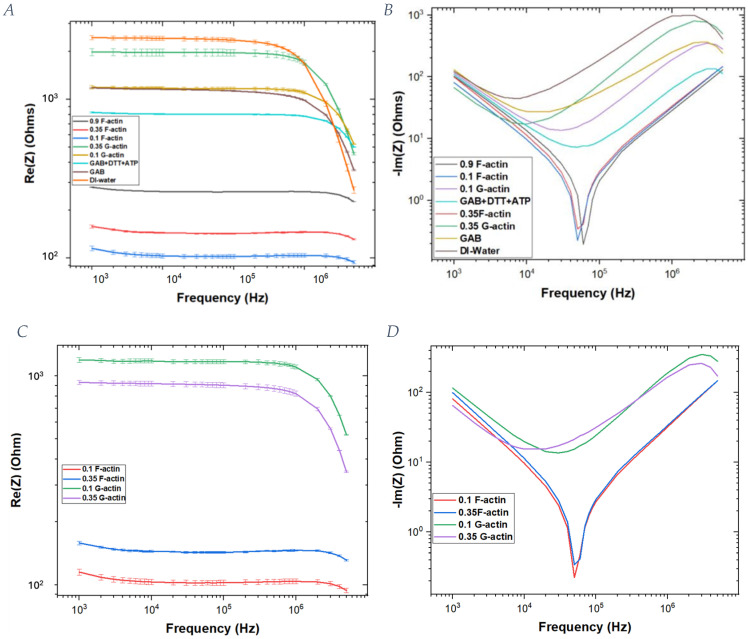
Real and imaginary parts of the impedance of G-actin, F-actin and buffer solutions. (**A**) Real part of all the samples. (**B**) Imaginary part of all the samples. (**C**) Focus on the real part of G-actin and F-actin at different concentrations. (**D**) Focus on the imaginary part of G-actin and F-actin solutions.

Subsequently, the impedance zeta has been split into its real and imaginary parts (Figure 3). 

**Figure 4:**
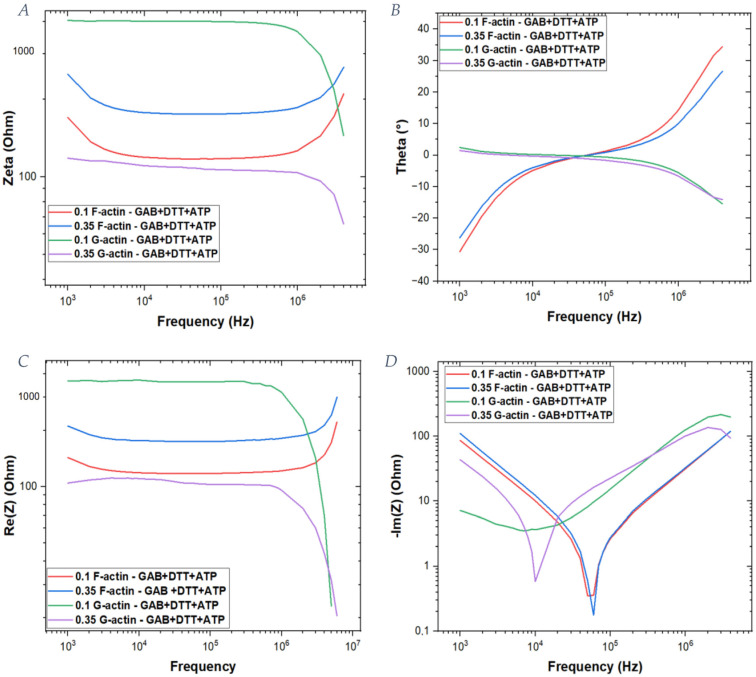
Subtraction of the background data from the sample’s solutions: general actin buffer (GAB), dithiothreitol (DTT) and adenosine triphosphate (ATP). (**A**) Impedance of the background-subtracted samples, comparing G-actin and F-actin solutions. (**B**) Phase of the background-subtracted samples. (**C**) Real part of the impedance. (**D**) Imaginary part of the impedance.

The curves in Figure 4 represent the actin solution sample values from which the background (the buffer solution itself) has been subtracted. Since ionic currents must traverse both the solution and actin monomers as they move to the opposite electrodes, the globular actin is modeled as a circuit element in series with the media. Instead, actin filaments are assumed to be in parallel with one another and with the media as well. Thus, different calculations were made to obtain the final graphs. 

Table 1 reports data for the actin filaments as well as for the globular actin in the media at the different actin concentrations tested.

Such data have been used to extrapolate Figure 3. Additional data are reported in the Supplementary Materials (Figures S1–S17), from which it is clear that there is only a small standard deviation involved in the reported values and a significant difference between globular and filamentous actin. 

For completeness, in Table 2 we summarize the three independent electrical contributions (*R*, *C* and *L*) to the sample’s impedance at two different concentrations of F-actin.

## 3. Discussion

Exploring the results of our measurements, the conditions that allow actin filaments to behave as electrical transmission lines for ion fluxes along their lengths were investigated. Actin filaments may contain a proportion of their surrounding counterions in the form of a condensed cloud around their length [12], and this is dependent on both the dielectric constant of the surrounding medium and the ionic concentration of the solution. Further, whereas all monomers of globular actin contained within their filaments have tight binding and form a double-stranded helix, the distribution of counterion clouds is nonuniform along the filament’s length due to an inhomogeneous charge distribution on F-actin. Thus, it is reminiscent of a solenoid with oscillating currents flowing along and around it due to voltage differences generated by the ends of filaments. However, the solenoidal-like flow of ions requires a proper geometrical orientation of the electric-field-driven ionic flows and the alignment of actin filaments along these lines. Otherwise, the ion flows would be linearly oriented, undergoing possible collisions with actin, which would impede their movements. 

In general, this cloud of counterions gives F-actin resistive, capacitive and inductive characteristics that are associated with a highly conductive medium: the inductive component of the electrical properties of ionic waves is due to actin’s double-stranded helical structure that induces the ionic flow in a solenoidal manner [24]. The ion flow is expected at a radial distance from the center of the filament, which is approximately equal to the Debye length, and this distance depends on the ionic concentration of the medium. 

Three samples containing solutions of actin filaments in the buffer have been used with the following protein concentrations: 2 μM, 7 μM and 18 μM. The average length of the filaments is estimated to be ~8 µm as described in the protocol in the Materials and Methods section, considering each monomer to be 5.5 nm in length. Hence, it is possible to calculate the number of actin monomers in one filament of the helix (N_1_ ~1455) and the total number of actin monomers in a double-stranded filament (N_2_ ~2910). 

In the case of the 2 µM actin concentration, considering the chamber’s volume of 2.625 µL for the conductance measurement and the molecular weight of actin monomers, the total number of actin filaments in the chamber is estimated to be around ~1.09 × 10^9^, and the number of actin monomers is approximated as ~3.16 × 10^12^. For the 7 µM actin sample, the total number of filaments in the chamber appears to be ~3.80 × 10^9^, and the number of monomers is estimated to be ~1.11 × 10^13^. Finally, for the 18 μM concentration, the number of filaments is found to be ~9.78 × 10^9^, while the number of monomers is estimated as ~2.85 × 10^13^.

Our goal was to find an accurate electric circuit model composed of capacitors, resistors and inductors to explain the observed electrical behavior of the actin filaments in the medium. From the figures shown above (Figure 2, Figure 3 and Figure 4), a substantial difference between actin filaments and globular actin monomers can be seen. Considering the zeta impedance reported in Figure 2, we can infer that G-actin has a similar trend to the buffers, and it has a higher impedance than the corresponding actin filaments. Moreover, considering impedance as the reciprocal of conductance, it is possible to conclude that actin filaments have higher conductance than G-actin under the same conditions of ionic strength and protein concentration. 

Both the total resistance and capacitance of the system have been calculated using the formulas reported below and the data in Table 1 and Table 2. The capacitance has been calculated using the lowest frequency available (*f_i_* = 1 kHz).

(1) $$Z=\sqrt{{Re}^{2}+{Im}^{2}}$$

(2) $$\tilde{R}=Re\left(Z\right)\;$$

(3) $$\tilde{C}=\frac{1}{Im\left(Z\right)*{2\pi f}_{i}}\;$$

Calculations have been also performed for the contribution to capacitance and resistance due to the medium itself, considering first only GAB, then GAB plus ATP and DTT. In the case of GAB alone, the capacitive contribution is estimated to be ~1.2 × 10^−6^ F and the resistive contribution ~1.2 × 10^3^ Ω. In the second case (GAB + ATP + DTT), the capacitive contribution is ~1.5 × 10^−6^ F, and the resistive is ~8.3 × 10^2^ Ω. Subsequently, the influence of the media is conveniently subtracted to calculate the electrical element characterizing both unpolymerized and polymerized actin. 

To this end, the following formulas have been used to calculate the effective impedance of the total filament component and both the relative real and imaginary parts. These data were used to extrapolate curves shown in Figure 4.

(4) $$Z_{f}=\frac{1}{\frac{1}{Z_{f+m}}-\frac{1}{Z_{m}}}=\frac{1}{\frac{Z_{m}-Z_{f+m}}{Z_{f+m}*Z_{m}}}=\frac{Z_{f+m}*Z_{m}}{Z_{m}-Z_{f+m}}$$

(5) $$Re(Z_{f})=\frac{\left[\left(x^{2}+y^{2}\right)*u+\left(u^{2}+v^{2}\right)*x\right]}{\left(u^{2}+v^{2}\right)-\left(x^{2}+y^{2}\right)}$$

(6) $$Im(Z_{f})=\frac{\left[\left(x^{2}+y^{2}\right)*v+\left(u^{2}+v^{2}\right)*y\right]}{\left(u^{2}+v^{2}\right)-\left(x^{2}+y^{2}\right)}$$

$$with\;x={Re}_{f+m};\;y={Im}_{f+m};\;u={Re}_{m};\;v={Im}_{m}$$

Using these curves, it is possible to observe that the formed filaments have a very low impedance (~10^2^ Ω) that increases when the actin concentration is increased. On the other hand, for the G-actin solution, the impedance is 10 times greater, and it increases with actin concentration. Additionally, there is a difference in the trend of the curves: G-actin solutions have a constant impedance up to a frequency of 10^6^ Hz, whereas in the case of F-actin at low frequencies, there is a decrease in the impedance values; then, a constant value persists up to 10^6^ Hz, above which an exponential increase occurs as the frequency further increases. Moreover, the behavior of the phase shows differences when making a comparison between G-actin and F-actin solutions. In particular, actin filaments are characterized by a peak at which the imaginary part approaches a zero value (10^5^ Hz), which is clearly different from the globular actin’s phase trend.

The capacitive and resistive contributions of a single actin monomer and filament were then estimated and compared to previous theoretical predictions. To determine if these values were consistent with theoretical predictions depending on the ionic concentration, the total capacitance of a single filament has been calculated by previous models [31] according to the formula

(7) $$C_{fil,theor}=\frac{2\pi \varepsilon l}{\ln(1+\frac{\lambda_{D}}{r})}$$

where, *l* is the length of the filament, *r* is the radius of the actin monomer, *ε* is the absolute permittivity of the solution, and *λ_D_* is the Debye length given by Equation (1) [19].

As introduced before, the Debye length depends on the ionic concentration of the buffer c*_s_*, which in our case is 500 mM. Under physiological conditions (approximately 100 mM), the Debye length is approximatively 0.8 nm [31]. Hence, positive counterion charges in the bulk are expected to form a cylinder with a radius greater than the actin filament itself, away from the actin surface, which includes the condensed ions.

(8) $$\;\;r_{actin}<r<r_{actin}+\lambda_{D}$$

In the present study, more realistic assumptions were adopted, namely that the length of an actin filament was 8 μm and its radius was taken to be 2.75 nm. The theoretical capacitance for a single actin filament was then found to be *C_fil_theor_* = 6.97 × 10^−3^ pF. 

Using the total number of actin filaments in the measurement chamber as 1.09 × 10^9^ and 3.80 × 10^9^, in the case of 2 μM and 7 μM, respectively, and assuming the filaments to be in parallel with respect to one another, the capacitance of a single filament at these two concentrations is extrapolated to be

$$C_{\mathrm{fil}2}={\tilde{C}}_{f}/N_{\mathrm{fil}2}=1.65\;\times \;10^{-3}\;\mathrm{pF}\;\;\;\mathrm{and}\;\;\;C_{\mathrm{fil}7}={\tilde{C}}_{f}/N_{\mathrm{fil}7}=3.68\;\times \;10^{-4}\;\mathrm{pF}$$

There is a discrepancy of a 4.48 factor between these two results, which may be due to the formation of irregular networks of actin filaments other than parallel arrangements. 

Thus, with these assumptions, our results for the 2 μM actin concentration are found to be four times lower than the calculated theorical capacitance (*C*_fil2_ = 1.65 × 10^−3^ pF), while in the case of the 7 μM actin solution, we have obtained a nineteen times lower value (*C*_fil7_ = 3.68 × 10^−4^ pF) than the previous theoretical estimate. This is summarized below:

$$C_{fil2}\approx \frac{C_{fil\_teor}}{4};\;\;\;C_{fil7}\approx \frac{C_{fil\_teor}}{19}$$

However, this discrepancy may be again explained as a result of the oversimplified assumption that all actin filaments in solution are in parallel with one another. As the concentration of actin in solution increases, this divergence increases in proportion to the concentration increase, indicating that actin filaments may be forming differently connected networks or may even be undergoing annealing to make longer filaments on average by joining two or more filaments end to end. Further, it is possible that they could also form series connections between one another in addition to parallel connections or forming even more complex network structures. Moreover, in the F-actin solution, some globular actin could remain unpolymerized, contributing to the formation of series connections. Additionally, our assumption about the average length of an actin filament is not necessarily realistic, with a Gaussian probability distribution of lengths around the average being closer to the actual situation.

On the other hand, the actin monomer cannot be modeled by the previously assumed cylindrical capacitance because it is not comparable to a cylinder but has an almost spherical geometry instead. The formula for the capacitance of a spherical capacitor is given by

(9) $$C_{g,theor}=4\pi \varepsilon \frac{r\left(r+\lambda_{D}\right)}{\lambda_{D}}$$

The permittivity, *ε*, is given by *ε* = *ε*_0_*ε_r_*, where *ε_r_* is the relative permittivity, which should be in a range between 3 < *ε_r_* < 10 for an ionic solution surrounding actin. We hypothesize that with a value of *ε_r_* = 4 being assumed as a typical value for an organic material such as actin, a reasonable estimate can be obtained. The length of an actin monomer is approximately 5.5 nm, and the radius of the actin filament is *r_actin_* = 2.5 nm [24]. Consequently, the capacitance for a single actin monomer has been approximately determined to be 96 × 10^−6^ pF using theoretical considerations [24]. This value was obtained by using Equation (7), substituting *ε_r_* = 80 and with the Bjerrum length replacing the Debye length. 

According to such calculations [24], a higher value for the globular actin capacitance was expected compared to the currently obtained experimental results. The globular actin components were assumed to be in parallel with one another and in series with the media, and Equation (9) was used to calculate the theoretical capacitance of a single monomer. As previously stated, the total number of actin monomers measured in the chamber was 3.16 × 10^12^ in the 2 μM solution and 1.11 × 10^13^ in the 7 μM solution, with a monomer’s radius of approximately 2.5 nm. Considering *λ_D_* = 0.8 nm and *ε_r_* = 4, based on the assumptions made above, the theoretical value for a single monomer of actin is found to be C_g,theor_ = 4.59 × 10^−6^ pF, which is one order of magnitude lower than the value calculated earlier [24]. We then divided the total capacitance of G-actin solutions in two different concentrations by the number of actin monomers present in each chamber. In the first case, the experimental capacitance of a single monomer is found to be equal to 4.43 × 10^−7^ pF (at 2 μM), while in the second case, it is found to be equal to 2.16 × 10^−7^ pF (at 7 μM). The values obtained are around 10 and 21 times lower than the previous theoretical predictions.

Regarding the resistive contribution to be compared to the experimental values, the following equation was used for theoretical predictions:

(10) $$R=\frac{\rho \ln\left((r_{act}+\lambda_{D}\right)/r_{act})}{2\pi l}$$

Considering ρ = 0.82 (Ωm), the theoretical resistance for a single 5.5 nm long monomer results to be 6.7 MΩ [31], which is consistent with the experimental results. Extrapolating these assumptions to one actin filament, the theoretical value of its resistance is evaluated as 9.75 GΩ (considering a length of 8 μm). As regards the resistance of a single actin filament (8 µm long) in both 2 µM and 7 μM solutions, the total resistance of the F-actin meshwork is given by $\tilde{R}$_f_, while each filament has resistance *R*_fil2_ = $\tilde{R}$_f_ × N_fil2_ = 1.56 × 10^11^ Ω and *R*_fil7_ = $\tilde{R}$_f_ × N_fil7_ = 7.99 ×10^11^ Ω. The two estimates are within 19.5% of each other, which is reasonably close. However, this should also be compared to the previous computational estimates.

Previous theoretical estimates have been made [24] for a 1 μm filament, and when extrapolated to the current case of 8 μm filaments, they result in a value of *R*_theor_ = 8.73 × 10^9^ Ω, which is two orders of magnitude lower than our experimental estimates. However, again, formation of more complex networks in the sample could readily account for this difference. For example, if we dealt with an array of n_1_ rows and n_2_ columns of resistive elements in a 2D matrix so that N = n_1_ × n_2_, then the approximate values of each filament’s resistance (assuming all being equal) would be *R*_fil_ = $\tilde{R}$_f_ (n_2_/n_1_) instead of *R*_fil_ × N if all of them were in parallel.

Another important aspect to be analyzed is the inductive contribution of the actin filaments. In particular, we assume that this is due to the actin’s double-stranded helical structure that induces an ionic flow with a solenoidal geometry. It is well-known that the inductance does not contribute at low frequencies but it does at high ones. Inductance can be obtained from the resonant frequency *f*_0_, at which the imaginary part of Z tends to zero as

(11) $$L=\frac{1}{C*({2\pi f}_{0}{)}^{2}}$$

Table 2 summarizes the resistive, capacitive and inductive contributions of the solutions with actin filaments at different concentrations, subtracting the background data, as previously explained. Based on Table 2 and using Equation (11), the value of the inductive contributions of the actin filament solution at 7 μM is *L* ~4.9 × 10^−6^ H; at 2 μM, it is *L* ~4.5 × 10^−6^ H. In the first case (2 μM solution), the corresponding frequency f_0_ to the resulting peak is 55 × 10^3^ Hz; in the second case (7 μM solution), *f*_0_ is ~60 × 10^3^ Hz. Near the resonant frequency of the applied AC electric field, the inductive and capacitive contributions have the same order of magnitude in the case of actin filament ensembles measured. This provides support to the assumption made on the solenoidal shape of the filaments and their condensed clouds of counterions in their surroundings, contributing to the inductive behavior of these nanowires.

We then compare the experimental results with theoretical predictions to determine their consistence with the following standard formula for a solenoid:

(12) $$L=\frac{\mu N^{2}A}{l}$$

where *l* is the length of the F-actin, *N* = a/r_h_ is the number of turns the ionic current makes around the filament when tightly wound up, and A is the cross-sectional area of the effective coil. In previous studies, considering the magnetic permeability of the medium, the inductance calculated using Equation (12) was found to be *L* = 1.7 pH for the length of the monomer [32]; such a value, can be extrapolated to a corresponding estimate for an 8 μm long actin filament to yield *L* = 1.2 × 10^−15^ H = 1.2 fH. It should be noted that this is a maximum theoretical estimate, assuming ions are moving in a solenoidal manner around and along the AFs and being the most tightly packed possible.

To make a comparison with the above theoretical results, the inductive contributions of a single actin filament were calculated from experimental data obtained in this study, assuming the filaments to be in parallel with one another. The inductance of a single filament at the two concentrations is, respectively, L_fil2_ = L_f_/N_fil2_ = 4.12 × 10^−15^ H and L_fil7_ = L_f_/N_fil7_ = 1.32 × 10^−15^ H. These values are very close to the maximum theoretical predictions for the inductance of an 8 μm long AF, especially in the case of the higher concentration of actin, which is only about 10% off. This relatively small discrepancy can be explained by at least two factors: the first involves the numbers of turns around the solenoid that moving ions make in the process; the other factor involves the geometrical distribution of AFs, which is most likely a combination of parallel and series arrangements. Finally, the predicted solenoidal ion motion around AFs assumes that the ions are flowing in solution driven by electric fields oriented approximately parallel to the AF filaments. This can be the case only for a proportion of the filaments as they are a priori oriented randomly with respect to the electrodes, although they may rotate to be along electric field lines over time.

The idea of modeling actin, both in its globular and filamentous form, as an RLC circuit is widely supported by the literature on this topic [7,26,33]. This type of model has been implemented in computational analysis, and it has undergone subsequent optimization, as shown in detail in the work of Marucho et al. [25,27,28]. Furthermore, the RLC circuit representation for actin has been extensively discussed and applied to actin in specific biological contexts by the group of Satarić et al. [34,35], as originally proposed by Luxford et al. [24], providing support for its validity.

**Figure 5:**
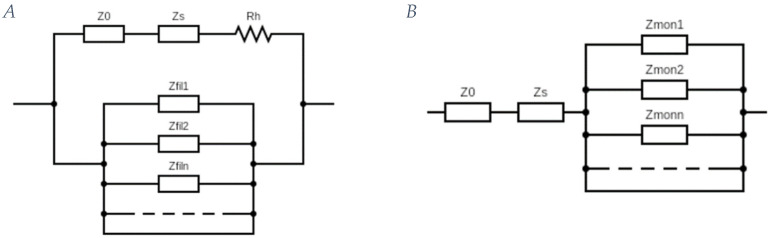
(**A**) Model of actin filaments in parallel with the media. Z_0_ is the impedance of the external elements, Z_s_ is the impedance of the solutions, R_h_ is the constant resistance representing the small residual fraction of unpolymerized globular actin, and Z_filn_ is the total impedance of the nth filament; (**B**) model of globular actin in series with the media. Z_monn_ is the impedance of the nth monomer; Z_0_ and Z_s_ as for Figure (**A**).

A possible scheme of these circuits is represented in Figure 5, where Z_0_ is the impedance of the external elements, Z_s_ is the impedance of the solutions, R_h_ is the small constant resistance that represents the small residual fraction of unpolymerized globular actin, Z_filn_ is the total impedance of the nth filament, while Z_monn_ is that of the nth monomer.

To further elaborate on the meaning of the curves obtained by changing the frequencies in our study, we hypothesized that depending on where we are on the frequency axis, we could fall into the condition of percolated resistors or percolated capacitors [32]. Related previous research investigated conduction flow pathways through disordered random composite media in terms of effective medium approximation (EMA), RC networks and the frequency-dependent admittance of large two-dimensional square resistor–capacitor networks [32,36,37,38]. Therefore, exploiting our experimental data, the solutions of both G-actin and buffers can be seen as RC networks with percolated resistors, exhibiting several similar features to the theoretical admittance and phase represented by the earlier models. On the other hand, the actin filaments seem to behave differently. In particular, the trend seen in the phase reminds one of a combination of an RC circuit characterized by percolated resistors at low frequencies but percolated capacitors at high frequencies. Regarding the admittance, such experimental curves for actin filaments in solution did not show any similarities with the theoretical predictions. The reason may be related to the presence of the inductive contribution that we found to exist in the experimentally investigated buffer solutions in contrast to the theoretical model that did not include inductors in the network.

Finally, it is instructive to compare the estimates for the *R*, *C* and *L* obtained for a single actin filament with those extrapolated in an earlier publication for microtubules [39,40]. For the sake of comparison, we assume both types of filaments to be 20 μm long. For microtubules, the estimates have been made based on experimental and theoretical bases, giving *R* = 2 GΩ, *C* = 2 pF, and *L* = 7 fH. For actin filaments, the results of the present papers give the following values for an actin filament of the same length as that of a microtubule: *R* = 24.4 GΩ, *C* = 4.1 fF, and *L* = 1.3 fH. We can conclude that comparable size actin filaments are more resistant to ion flows and have a vastly lower capacitance and somewhat lower inductance. These results are consistent with the geometrical differences between the two types of protein filaments and a large difference in the electrostatic charge density on their surfaces, making microtubules more conductive for ionic flows and also more capacitive as their surface area is much larger and the charge density is also much higher. Moreover, one could compare the resonant frequencies in these two cases with an estimate for 20 μm microtubules, indicating a resonant frequency *f*_0_ = 0.65 THz, while the same length actin filament would resonantly respond to a frequency of *f*_0_ = 69 THz. The quality factor in both cases is very large, suggesting a very broad range of frequencies around the resonant value and an overdamped case. However, recent experiments with both types of filaments are supportive of these estimates, with microtubules being destroyed by THz pulses between 0.5 and 1.5 THz [41], while actin filaments underwent a similar destructive process at 4 THz [42].

## 4. Materials and Methods

In total, 1 mg of unlabeled lyophilized actin powder (cytoskeleton actin protein (>99% pure): rabbit skeletal muscle Cat. # AKL99) was reconstituted in 0.333 mL deionized water (DI-H_2_O). Then, it was diluted by adding 2.165 mL of general actin buffer (cytoskeleton, general actin buffer Cat. # BSA01) and 5 µL of ATP (cytoskeleton, adenosine 5′-triphosphate disodium salt (ATP) Cat. # BSA04, 100 mM stock) was added to yield a final ATP concentration of 0.2 mM. Then, 12.5 µL of 100 mM DTT was added to yield a final concentration of 0.5 mM DTT in the actin solution, and the final solution was incubated on ice for 1 h. Centrifugation was carried out at 1400 RPM for 15 min at 4 °C. Then, the actin solution was aliquoted in 100 µL samples, and the polymerization reaction was assembled by adding $\frac{1}{10}$th of the volume of actin polymerization buffer (cytoskeleton, APB at 10X Cat. # BSA02). In total, 10 µL of APB followed by 1.1 µL of ATP were added to each aliquot. The surplus aliquots were snap frozen and stored at −80 °C. Once ATP was added to each aliquot, the polymerization reaction took place at room temperature for 1 h. Using this protocol, the average obtainable length of the filament has been determined as 8 µm.

Following these steps, samples of actin solution at different concentrations were obtained. Then, for testing the F-actin, three different concentrations of actin in buffer solutions were prepared for subsequent measurements—0.1 mg/mL, 0.35 mg/mL and 0.9 mg/mL, respectively—while for G-actin, only two solutions were reconstructed (with no polymerization), namely 0.1 mg/mL and 0.35 mg/mL. 

The concentration’s values were chosen following the scientific evidence on the optical actin concentration favoring the polymerization to be within the range 0.1 mg/mL–0.5 mg/mL [43,44,45]. For what concerns the 0.9 mg/mL one, it seems there is a slower polymerization when the actin concentration is greater than 0.5 mg/mL, so it was plausible to test a higher concentration for comparison.

### 4.1. Turbidity Measurements

Purified actin has been used to replicate actin polymerization in vitro so that the kinetics of the process can be studied. Turbidity measurements can be used to demonstrate and quantify the polymerization process of actin filaments. To this end, a spectrophotometer (SpectraMax^®^ iD5 microplate reader) and an optically clear vessel (96-well plate) were used. In total, 100 µL of the actin samples were quickly pipetted into the 96-well plate, and the turbidity measurement was performed with the following setting: 310 nm absorbance kinetic mode for 120 min at 25 °C [46]. Before each reading, the plate was shaken. In total, 241 points have been obtained, one every 30 s. If the protein Arp2/3 is added, the actin assembly is characterized by a short lag time, a period of net increase and a steady state (defined by a plateau); if not, it is characterized by an increasing straight line. Data were collected and analyzed using the Software OriginLab (KTE Interactive V9.1 Service Pack 7).

### 4.2. Electrical Conductance Measurements

**Figure 6:**
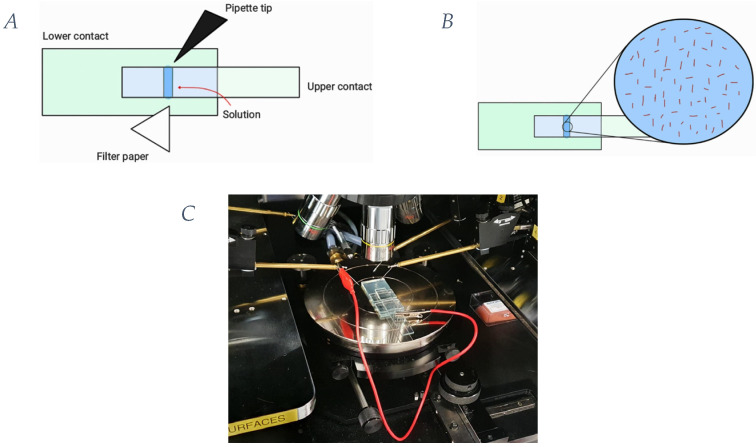
Experimental setup of the device configuration used to measure the impedance properties of actin filaments compared to the background. (**A**) Schematic representation of the device used. (**B**) Focus on the schematic representation of the actin filaments in solution in the perfused chamber of measurement. (**C**) Keithley 4200-semiconductor characterization system with Signatone probe station setup.

The building procedure for the electrical measurement device is reported as follows. Glass slides coated with fluorine-doped tin oxide (FTO) were used for each plate in the parallel-plate contact device (Sigma Aldrich, 735140, Merck KGaA, Darmstadt, Germany). Slides were cut with top contact dimensions of 1.5 mm × 10 mm × 50 mm and bottom contact dimensions of 1.5 mm × 27 mm × 50 mm. To eliminate surface particulates, slides were ultrasonically cleaned, and reactive ion etching (RIE) was performed exposing them to oxygen plasma (NGP80, Oxford Instruments, Abingdon-on-Thames, UK) for 5 min. A 70 μm thick double-sided tape was used to create a chamber 3 mm × 1.25 cm × 70 μm in size and a 3D-printed holder device was used to locate the upper electrode (Figure 6) [40].

Impedance spectroscopy in quasi-static mode on a Keithley 4200-semiconductor characterization system (SCS analyzer, Beaverton, OR, USA) equipped with a capacitance–voltage unit (CVU) and a Signatone probe station was used to collect the experimental data. The frequency was set within the range of 1 kHz–10 MHz.

CVU options were set, connecting the CV high potential cable to the collector number 4 and the CV low potential one to the collector number 1 (on the left). Copper alligator clips were used to bond the collector 1 and the upper glass of the parallel-plate device, whereas the common electrode was bonded to the lower glass. The setting included a frequency sweep and bias both were set to zero. The solutions were perfused into the experimental chamber of the parallel-plate contact device, which was connected to the Keithley by a micropipette tip at one opening and a paper filter at the other one for sucking eventual excess. At each data collection, the chamber was washed with a buffer, the general actin buffer (GAB). Any bubbles in the chamber should be avoided during the measurements; if they are present, they must be eliminated, and the measurement repeated to avoid any alterations in the results.

The analyzed solutions had three different concentrations in the case of actin filaments: 0.1 mg/mL, 0.35 mg/mL and 0.9 mg/mL; for globular actin solutions, there were two different concentrations: 0.1 mg/mL and 0.35 mg/mL. Buffers, such as GAB and GAB, plus both ATP and DTT were also added.

## 5. Conclusions

Following a successful reconstitution and polymerization of actin filaments, we explored the conditions that allow actin filaments to operate as electrical transmission lines for ion fluxes along their lengths. We reported a series of measurements under various conditions that enable us to make estimates of the electrical properties of actin filaments and globular actin under the present conditions. In the model used in this study, we followed earlier publications in this area and represented each actin monomer as an electrical element with both capacitive and resistive properties due to the actin filament’s molecular structure and the solution’s viscosity. In addition, the inductive behavior of actin filaments is associated with their double-stranded helical structure that allows the presence of ionic fluxes to propagate in a solenoidal manner. Regarding the capacitive contribution for a single actin filament, the experimental results are consistent with the theoretical predictions. For the 7 μM sample measurements, we expected a higher value, but this could be an effect of a more complex organization of the filaments in the solution when the protein is present at higher concentrations, in which case the filaments could also form series connections between one another. Moreover, some globular actin may have not polymerized completely, contributing to the series connections.

On the other hand, regarding the G-actin solution, assuming the globular actin monomers as spherical elements to be in parallel and comparing their capacitive contribution to theoretical estimates, our results appear to be consistent with the model. The admittance and phase curves of our samples’ measurements were compared to those in the literature on the conduction flow pathways through disordered random composite media and frequency-dependent admittance of large two-dimensional square RC networks. It can be concluded on this basis that both the G-actin and buffer solutions can be described as RC networks with percolated resistors, which is consistent with previously published theoretical predictions. Actin filaments appear to behave differently than globular actin. Regarding the phase, the trend resembles a combination of an RC circuit with percolated resistors at low frequencies and percolated capacitors at high frequencies, but no strong similarities between our experimental curves for actin filaments in solution and the admittance curves have been identified. This discrepancy could be related to the role of the inductive contributions present in our experimental samples and absent in the theoretical models. This is in addition to an increase in the protein concentration in the solution possibly explaining percolated behavior so that R, L and C elements form networks linking individual elements in a complex geometrical manner. Moreover, there is a significant number of filaments in the sample, so a major effort would be required to improve the model, but it would also involve a massive amount of image analysis and complex electrical network simulations. This should clearly be the focus of a future study but is outside the scope of the present paper.

## Figures and Tables

**Table 1:** Total capacitance and resistance contributions for solutions of actin filaments and globular actin at two different protein concentrations in the media, with and without the buffer contribution, respectively.

	F-Actin		G-Actin		F-Actin Buffer		G-Actin Buffer	
	$\tilde{C}$ (F) ^1^	$\tilde{R}$ (Ω) ^1^	$\tilde{C}$ (F)	$\tilde{R}$ (Ω)	$\tilde{C}$ (F)	$\tilde{R}$ (Ω)	$\tilde{C}$ (F)	$\tilde{R}$ (Ω)
2 μM	1.6 × 10^−6^	115.2	1.4 × 10^−6^	1194.4	1.8 × 10^−6^	142.8	22.1 × 10^−6^	366.0
7 μM	1.9 × 10^−6^	158.4	2.4 × 10^−6^	932.3	1.4 × 10^−6^	210.5	3.8 × 10^−6^	1169.3

^1^ $\tilde{C}$ (F) and $\tilde{R}$ (Ω) respectively the total capacitance and resistance for G-/F-actin.

**Table 2:** Resistive, capacitive and inductive contributions due to actin filaments alone in solution at different concentrations.

	F-Actin Buffer		
	*R* (Ω)	*C* (F)	*L* (H)
2 μM	142.8	1.8 × 10^−6^	4.5 × 10^−6^
7 μM	210.5	1.4 × 10^−6^	5.02 × 10^−6^

## Data Availability

All data generated or analyzed in this study are included in the article and in the Supplementary Materials. The raw datasets used and/or analyzed in the current study are available from the corresponding author upon reasonable request.

## Supplementary Materials

The following supporting information can be downloaded at: https://www.mdpi.com/article/10.3390/ijms25105485/s1.
