# Supplementary material for: Measurement and Characterization of the Electrical Properties of Actin Filaments
Source: Int J Mol Sci. 2024 May 17;25(10):5485. doi: 10.3390/ijms25105485 (PMC11121962; doi:10.3390/ijms25105485)
Supplement: Supplementary file 1 [file ijms-25-05485-s001.zip › ijms-3008569-supplementary.pdf]

Measurement and Characterization of the Electrical Properties of Actin Filaments

Supplementary Materials

Turbidity Measurements for F-actin polymerization under various conditions

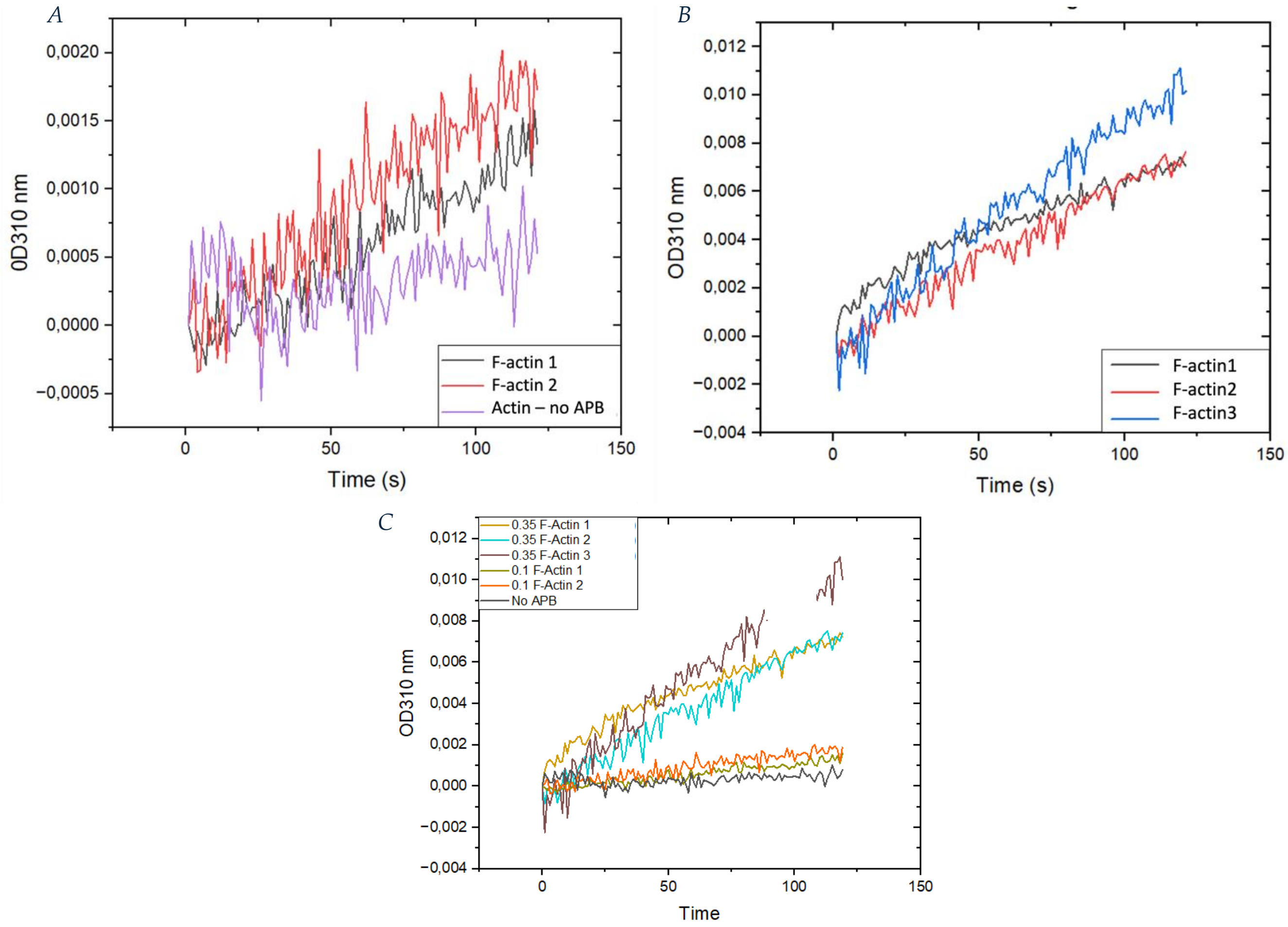

Figure S1. (**A**) Turbidity measurements of 0.1 mg/mL F-actin; (**B**) Turbidity measurements of 0.35 mg/mL F-actin; (**C**) Superposition of plots (A) and (B).

Impedance Measurements for F-actin under various conditions

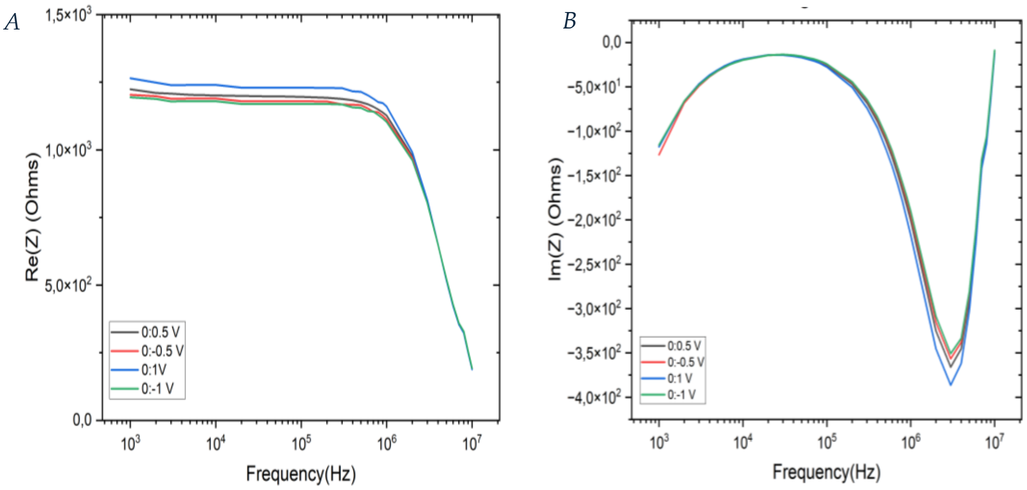

Figure S2. (**A**) Real part of 0.1 mg/mL F-actin; (**B**) Imaginary part of 0.1 mg/mL F-actin.

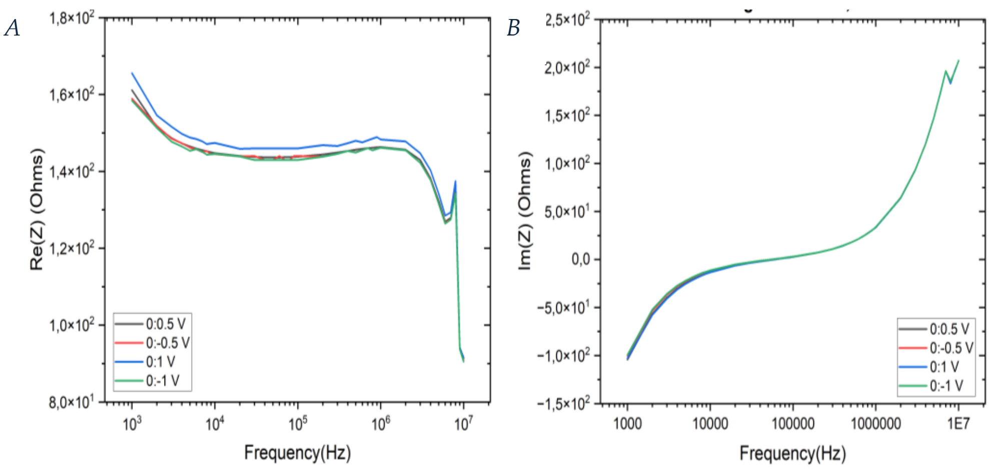

Figure S3. (**A**) Real part of 0.35 mg/mL F-actin; (**B**) Imaginary part of 0.35 mg/mL F-actin.

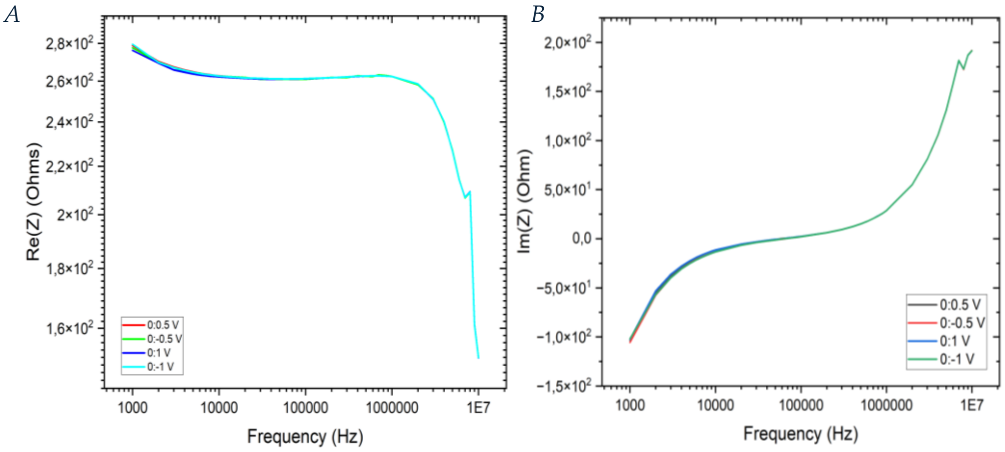

Figure S4. (**A**) Real part of 0.9 mg/mL F-actin; (**B**) Imaginary part of 0.9 mg/mL F-actin.

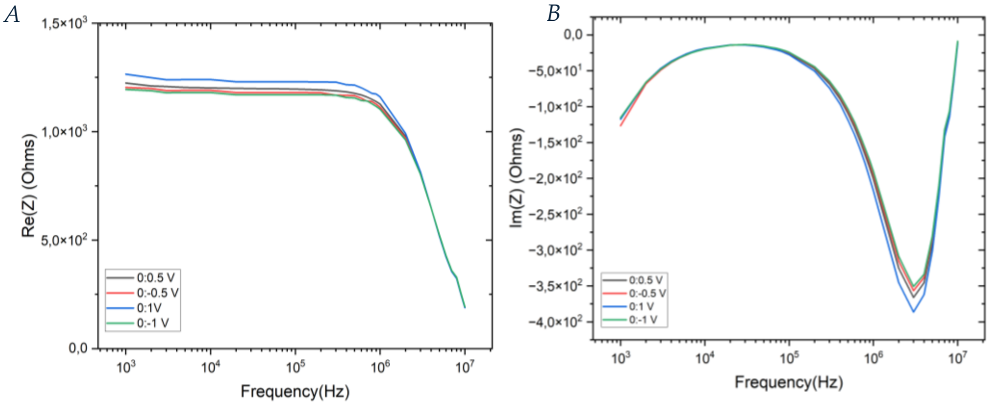

Figure S5. (**A**) Real part of 0.1 mg/mL G-actin; (**B**) Imaginary part of 0.1 mg/mL G-actin.

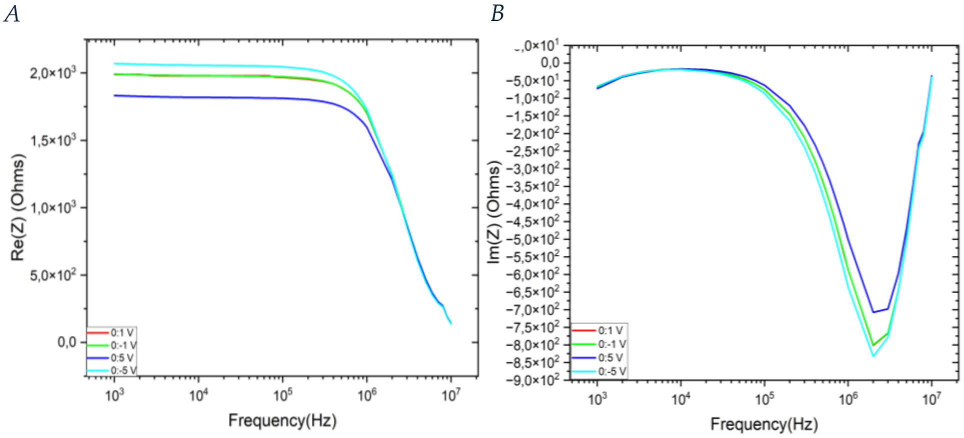

Figure S6. (**A**) Real part of 0.35 mg/mL G-actin; (**B**) Imaginary part of 0.35 mg/mL G-actin.

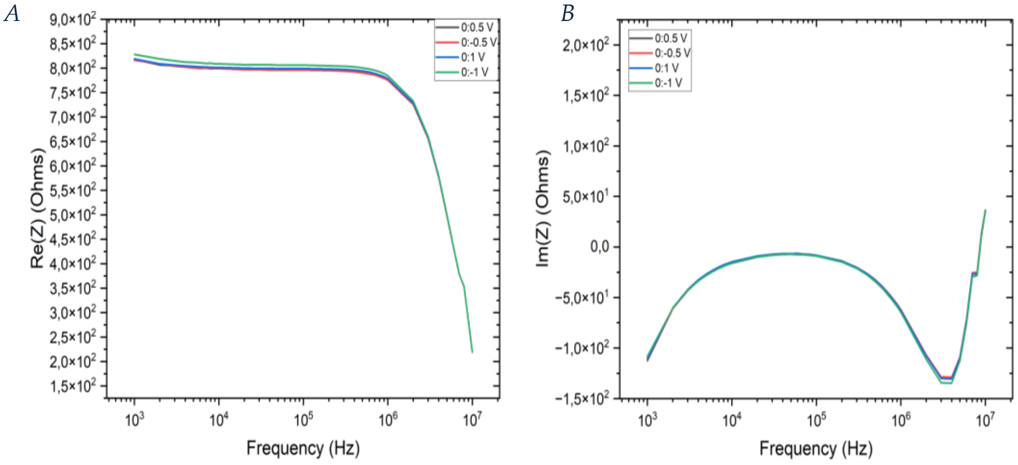

Figure S7. (**A**) Real part of GAB+DTT+ATP; (**B**) Imaginary part of GAB+DTT+ATP.

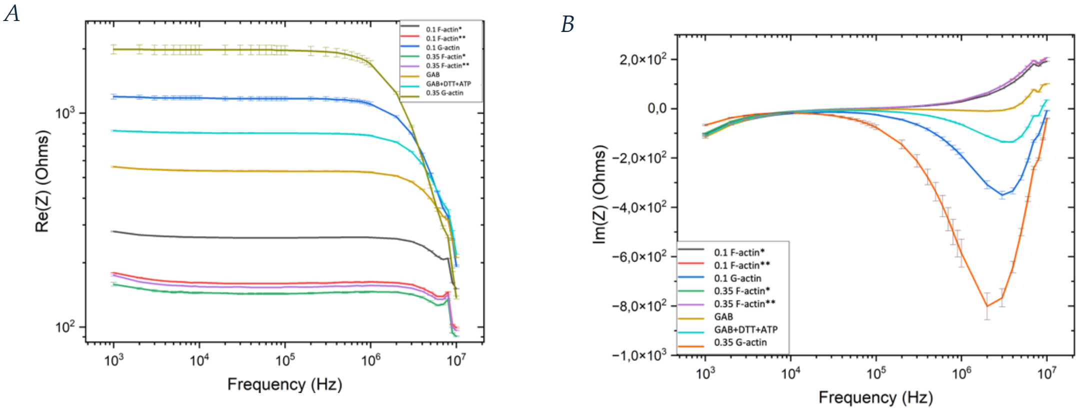

Figure S8. (**A**) Real part of G-/F-actin at different concentration; (**B**) Imaginary part of G-/F-actin at different concentration. If Rhodamine is inserted, then the legend contains two **; otherwise just one *.

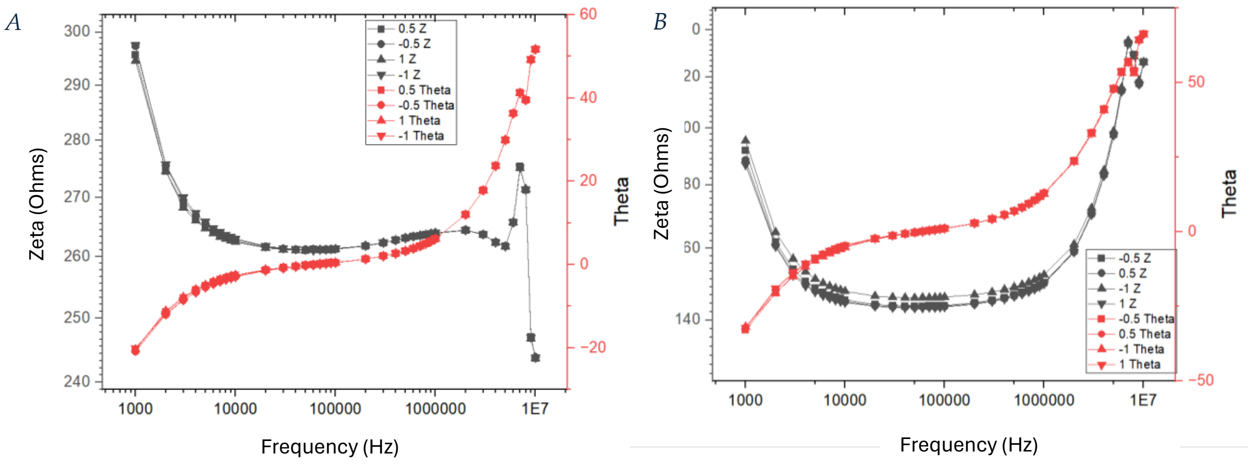

Figure S9. (**A**) Superposition of the real and imaginary parts of 0.1 mg/mL F-actin; (**B**) Superposition of the real and imaginary parts of 0.35 mg/mL F-actin. If rhodamine is inserted, then the legend contains two **; otherwise just one *.

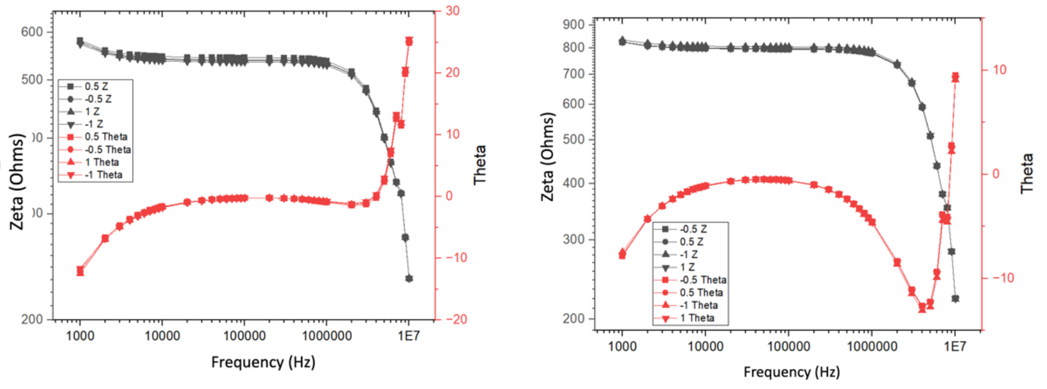

Figure S10. (**A**) Superposition of the real and impaginary parts of GAB buffer solution; (**B**) Superposition of the real and impaginary parts of GAB+DTT+ATP buffer solution.

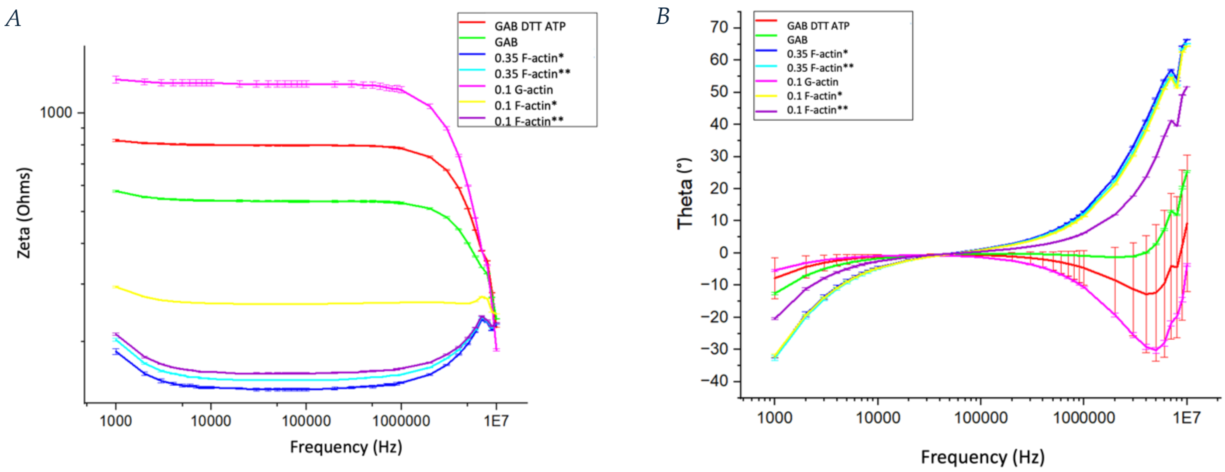

Figure S11. (**A**) Impedance (Zeta) reported for G-/F-actin and the buffers; (**B**) Phase (Theta) reported for G-/F-actin and the buffers. If rhodamine is inserted, then the legend contains two **; otherwise just one *.

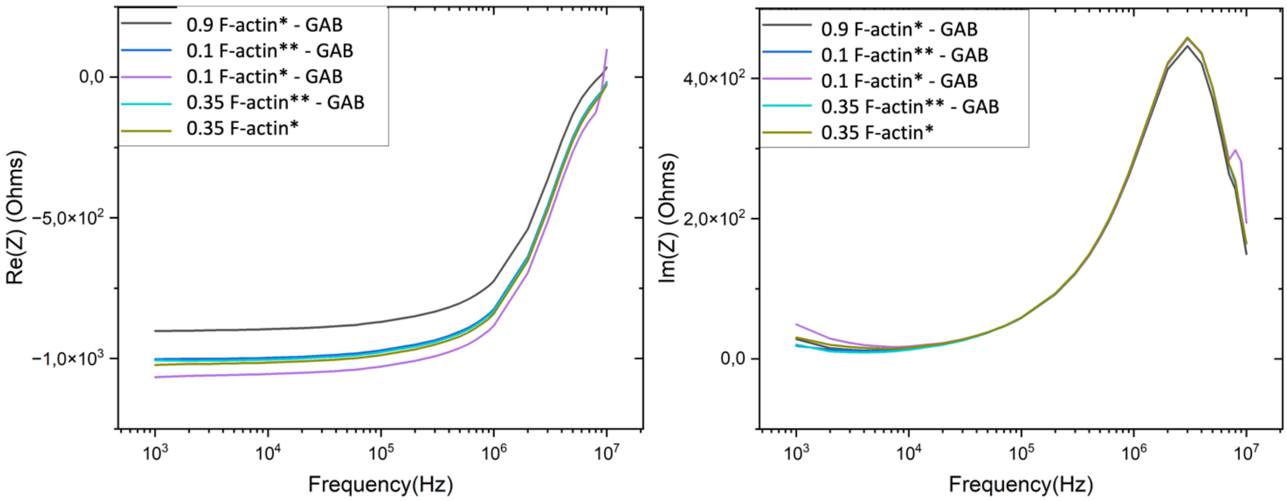

Figure S12. (**A**) Real part of F-actin minus the only contribution of GAB; (**B**) Imaginary part of F-actin minus the only contribution of GAB. If rhodamine is inserted, then the legend contains two **; otherwise just one *.

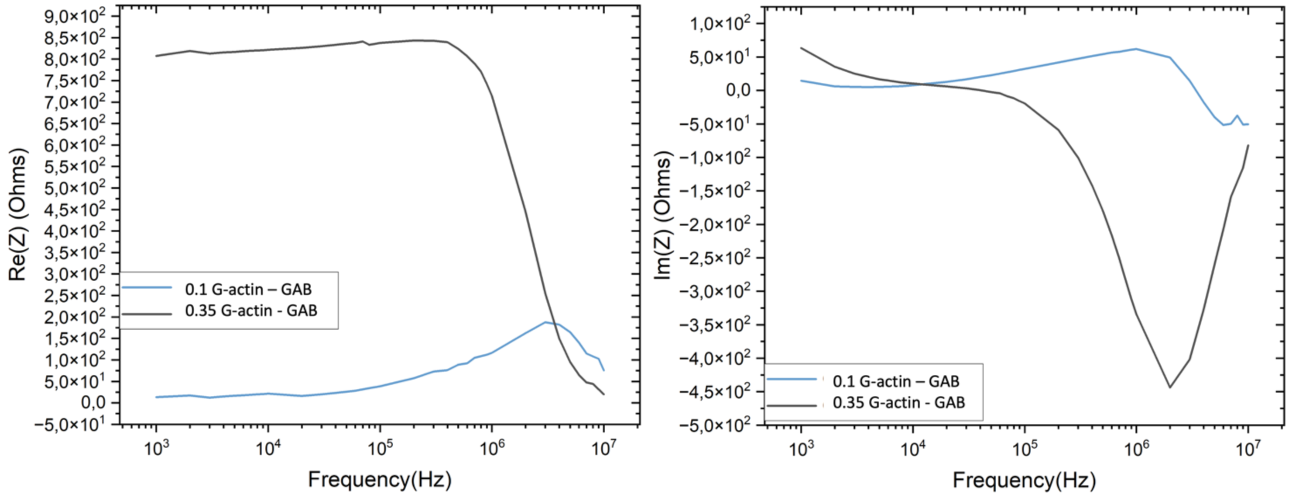

Figure S13. (**A**) Real part of G-actin minus the only contribution of GAB; (**B**) Imaginary part of G-actin minus the only contribution of GAB.

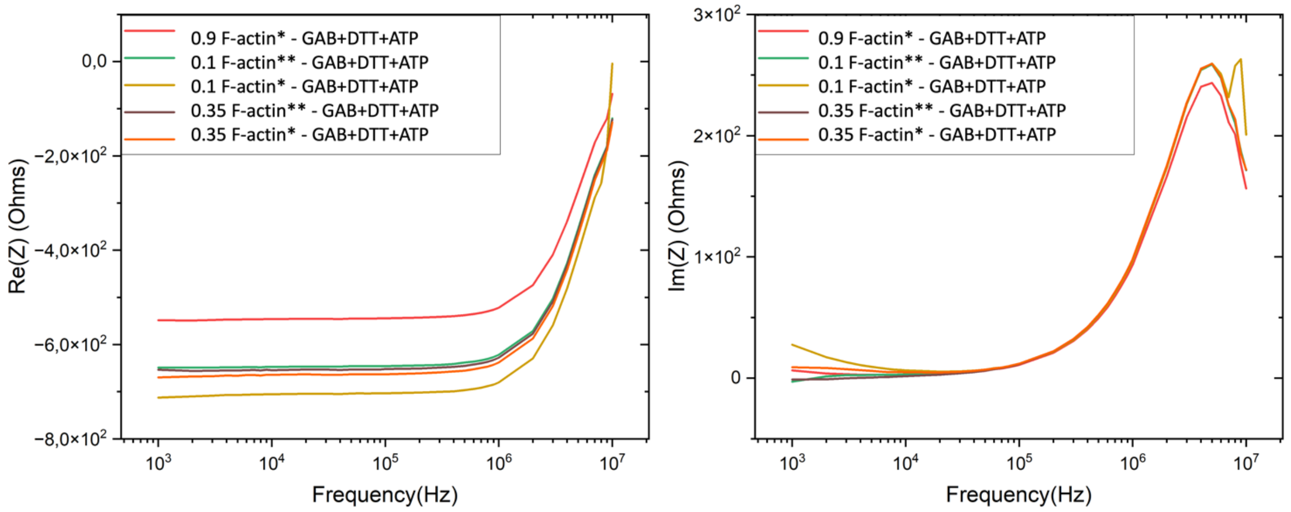

Figure S14. (**A**) Real part of F-actin minus the contribution of GAB, DTT, ATP; (**B**) Imaginary part of F-actin minus the contribution of GAB, DTT, ATP. If rhodamine is inserted, then the legend contains two **; otherwise just one *.

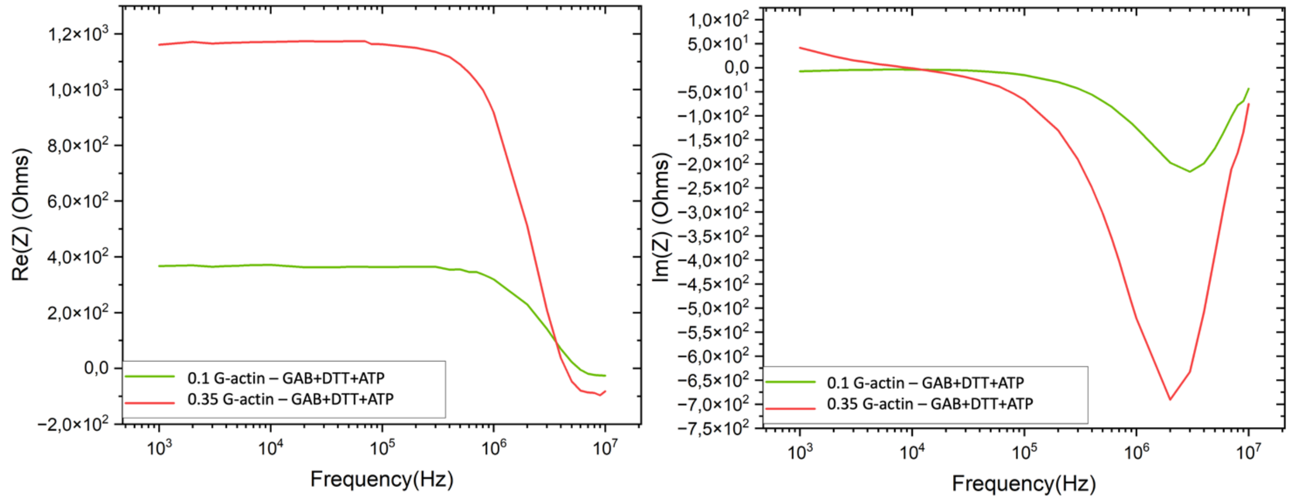

Figure S15. (**A**) Real part of G-actin minus the contribution of GAB, DTT, ATP; (**B**) Imaginary part of G-actin minus the contribution of GAB, DTT, ATP.

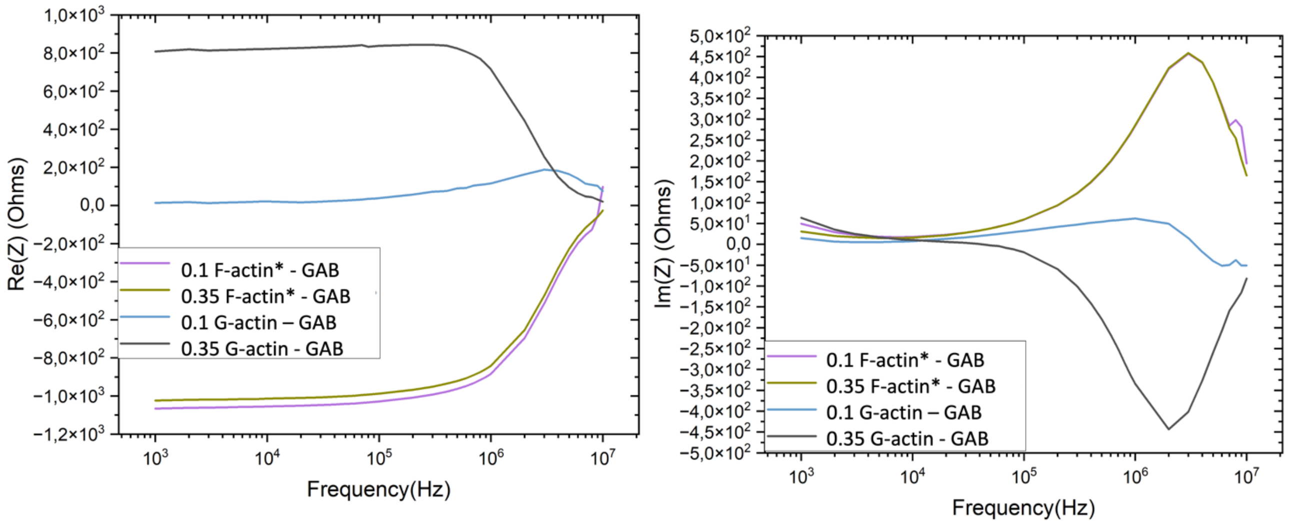

Figure S16. (**A**) Comparison of the real part of F-actin vs G-actin if only GAB is subtracted; (**B**) Comparison of the imaginary part of F-actin vs G-actin if only GAB is subtracted. If rhodamine is inserted, then the legend contains two **; otherwise just one *.

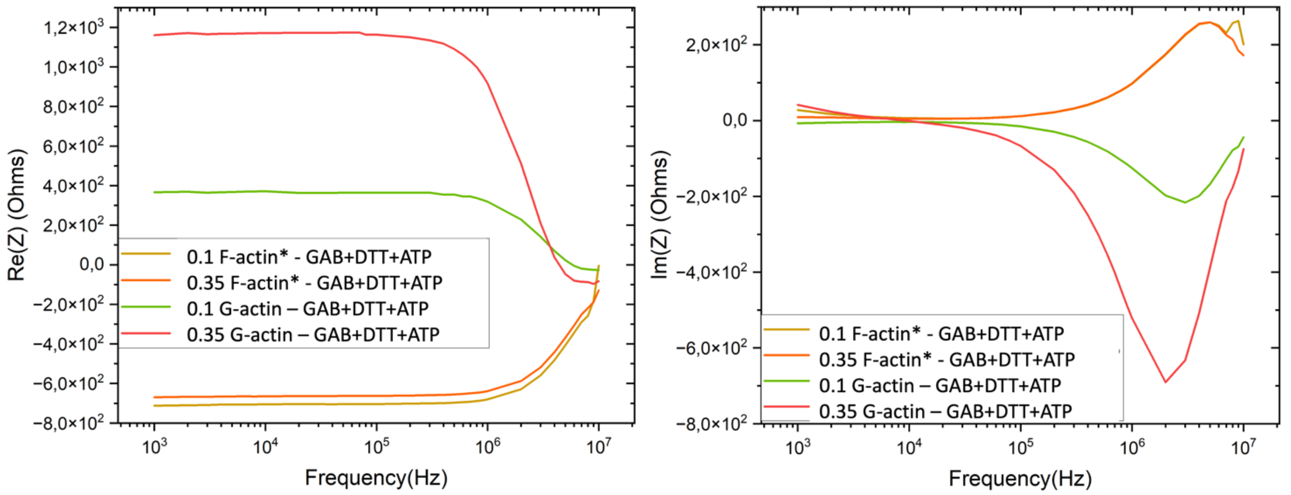

Figure S17. (**A**) Comparison of the real part of F-actin vs G-actin if GAB, DTT, ATP are subtracted; (**B**) Comparison of the imaginary part of F-actin vs G-actin if GAB, DTT, ATP are subtracted. If rhodamine is inserted, then the legend contains two **; otherwise just one *.
